# Could Transcranial Direct Current Stimulation Join the Therapeutic Armamentarium in Obsessive-Compulsive Disorder?

**DOI:** 10.3390/brainsci10020125

**Published:** 2020-02-23

**Authors:** Moussa A. Chalah, Samar S. Ayache

**Affiliations:** 1 Service de Physiologie, Explorations Fonctionnelles, Hôpital Henri-Mondor, AP-HP, 94010 Créteil, France; moussachalah@gmail.com; 2 EA 4391, Excitabilité Nerveuse et Thérapeutique, Université Paris-Est-Créteil, 94010 Créteil, France

**Keywords:** Obsessive-Compulsive disorder, OCD, transcranial direct current stimulation, tDCS, noninvasive brain stimulation, neuromodulation, treatment, therapy

Obsessive-compulsive disorder (OCD) is a mental disorder that can affect around 1–3% of individuals [1,2]. It is characterized by recurrent intrusive unwanted thoughts, ideas, urges, or images (i.e., obsessions) and/or repetitive mental acts or behaviors (i.e., compulsions) that occur in an attempt to neutralize obsessions, or according to rigid rules. Interestingly, various symptom dimensions of OCD could exist (e.g., symmetry, contamination, aggressive/sexual/religious obsessions); they might have specific neurobiological substrates and may be associated with different response to treatment [3,4]. OCD symptoms cause severe distress, drastically affect social, professional, or other relevant domains, and compromise the quality of life. OCD seems to have a bimodal age of onset (i.e., a first peak in late childhood/early adolescence and another peak in early adulthood) and a continuous symptomatology in most of patients, with a waxing and waning pattern observed in around 25% of patients [1]. 

Psychotherapy (i.e., cognitive behavioral therapy (CBT)) and psychopharmacology (i.e., selective serotonin reuptake inhibitor (SSRI)) are the available treatment regimen for this condition [1,2]. Among CBT interventions, exposure-response prevention is the most common one and consists of provoking obsessions by exposing the individuals to their obsession-triggering stimuli and enabling them to inhibit their avoidance behaviors or compulsions [1]. Moreover, in case of lack of insight or inability to tolerate such an exposure, cognitive-based interventions can be applied (i.e., cognitive reappraisal and restructuring) [1]. However, partial or no response seems to occur in a considerable part of patients following psychotherapy and pharmacotherapy administered in a monotherapy or a combined fashion. In these settings, and when switching to another SSRI or clomipramine fails, the available literature suggests testing augmentation strategies (i.e., adding an antipsychotic) or employing surgical techniques (i.e., neurosurgical ablation or deep brain stimulation of OCD brain circuitry), but the second option is reserved to severe treatment-resistant cases [1]. It is also important to keep in mind side-effects of the former (i.e., weight gain, risk of diabetes) and the latter (i.e., surgical adverse events). In this perspective, recent works have explored the potential efficacy of noninvasive brain stimulation (NIBS) techniques. 

NIBS techniques have been applied in several neuropsychiatric populations and consist of modulating the brain function by applying a magnetic field (e.g., transcranial magnetic stimulation) or an electric current (e.g., transcranial direct current stimulation (tDCS)) over the brain areas that are involved in the generation of a specific symptomatology. Several experimental and clinical studies have confirmed the safety profile of these techniques. In addition, the practicality, portability and relatively low cost of tDCS have allowed it to capture the attention of researchers worldwide, since it might serve as an alternative therapeutic tool if proven efficacious. tDCS consists of delivering a weak direct electric current (e.g., 1–2 mA) via a battery-driven stimulator connected to two saline-soaked electrodes (i.e., anode and cathode) applied over cortical areas of interest during a short time (e.g., 10–30 min) [5]. Neurophysiological studies employing motor evoked potentials have demonstrated that tDCS could lead to a subthreshold shift of neuron resting membrane potentials, in a way that anodal and cathodal tDCS, respectively, lead to depolarization and hyperpolarization. Pharmacological and imaging studies have suggested that tDCS induces local and connectional effects via synaptic and non-synaptic mechanisms, which could lead to modulating the regional activity of a cerebral target and/or its functional connectivity, as well as the oscillatory activities of specific networks [5]. Furthermore, the effects of this technique seem to depend on several variables, including the electrodes size, polarity (i.e., anodal vs. cathodal stimulation), and montage (cephalic vs. extracephalic montage), current flow direction, stimulation duration, protocol duration (i.e., single vs. multiple sessions), current intensity, as well as the nature (inhibitory vs. excitatory) and baseline activity of the stimulated neural networks, to cite a few [5].

Based on these data, tDCS might have its utility in the context of OCD where an underlying neural circuitry seems to be involved in the pathophysiology of this disorder. In fact, radiological studies have suggested abnormalities within a cortico-basal ganglia-thalamo-cortical loop that includes orbitofrontal-prefrontal areas, basal ganglia, thalamus, but also cerebellum, anterior cingulate cortex, insula, and temporal, parietal, and occipital regions [1,4]. In some of the studies that have adopted functional neuroimaging techniques, OCD was associated with abnormal activity in the orbitofrontal cortex (OFC), supplementary motor area (SMA), pre-SMA, and cerebellum [2,6,7,8,9,10]. Therefore, one may hypothesize that improving obsessive-compulsive (OC) symptoms could be possible by targeting these cerebral areas. In addition, the dorsolateral prefrontal cortex (DLPFC) is a carrefour for several cortico-subcortical networks, is involved in cognitive, emotional, and behavioral processes, and was chosen as a tDCS target in several neuropsychiatric symptoms [5]. Thus, targeting this area might also have its place in the case of OCD.

tDCS literature in the context of OCD includes case reports, open-label studies, and few randomized controlled trials [2,6,7,8,9,10]. Assessment included OC symptoms, by means of the Yale–Brown Obsessive and Compulsive Scale (YBOCS), as well as other comorbidities (i.e., anxiety and depressive symptoms). To start, the majority of case reports and open-label studies have adopted 10–20 tDCS sessions (20–30 min per session, 1–2 sessions per day) using a current intensity of 2 mA delivered by 25–35 cm^2^ electrodes [2]. YBOCS scores improved when targeting the DLPFC (anode over the left DLPFC, cathode over the right DLPFC/frontopolar region), OFC (cathode over left or right OFC; anode over contralateral cerebellar, occipital, cerebello-occipital, or parieto-temporo-occipital areas) and SMA or pre-SMA (cathode or anode over SMA/pre-SMA and reference electrode over the right OFC, occipital area, or deltoid) [2,6,7]. Conversely, cathodal stimulation of the left DLPFC (anode over the posterior neck-base) did not yield clinical benefits [2]. It is noteworthy that, regarding SMA or pre-SMA stimulation, OC symptoms seem to improve following cathodal stimulation; and improve or worsen following anodal stimulation [2]. However, the inconsistency in the effect of anodal tDCS might be related to difference in the electrode montage, which could impact the direction of the current flow. That is to say, clinical improvement and worsening/lack of change were respectively observed when using a cephalic (right OFC) versus an extracephalic (right deltoid) montage [2]. 

Besides case reports and open-label trials, a few randomized controlled trials have addressed this question. For instance, one double-blind randomized, controlled, partial crossover trial included 12 medicated patients with OCD who were non-responders to at least one pharmacological treatment (ten to twenty 20-min sessions, one session per day, current intensity: 2 mA, active electrode size: 25 cm^2^, reference electrode size: 35 cm^2^) [8]. Bilateral pre-SMA cathodal stimulation (anode over the right deltoid) resulted in a significant improvement in OC symptoms [8]. Such beneficial effects were not observed following bilateral pre-SMA anodal stimulation (cathode over the right deltoid). Although interesting, the results are limited by the absence of sham intervention. Another randomized parallel-arm sham-controlled trial in 25 SSRI-resistant medicated OCD patients documented beneficial clinical effects, following five consecutive days of anodal tDCS over the left pre-SMA (two 20-min sessions per day, current intensity: 2 mA, electrodes size: 35 cm^2^, cathode over right supra-orbital area) [9]. Beneficial tDCS effects documented in the two abovementioned studies might be due to the modulation of what is known as "the response inhibition", a key function of the pre-SMA, and by doing so tDCS could have enhanced patients’ capacity to suppress unwanted thoughts and behaviors. The inter-study difference in anodal effects might be due to the difference in tDCS montage (including cephalic vs. extracephalic reference), cohorts’ characteristics, sample size, and the presence/absence of sham condition. Interestingly, the right OFC might not be an inert reference, and therefore, it is not possible to formally eliminate effects emerging from cathodal stimulation of this area [9]. One may also wonder how bilateral cathodal and anodal tDCS over the pre-SMA could lead to significant clinical improvement. A more recent double-blind randomized sham-controlled study has adopted a parallel design (*n* = 10 in the active arm vs. *n* = 11 in the sham arm) [10]. The cathode and anode were placed over the left OFC and the right cerebellum, respectively, based on neuroimaging studies that showed OFC hyperactivity, and cerebellar hypoactivity or hyperconnectivity with basal ganglia in OCD patients [10]. Beneficial effects were obtained by applying ten 20-min tDCS sessions (twice per day, current intensity: 2 mA, electrodes size: 35 cm^2^) in medicated OCD patients with treatment-resistant profile. However, the effects were only observed immediately after the stimulation blocks, but not one month later. Dose-dependent tDCS effects have been observed in several clinical settings, and changing the tDCS parameters might enable obtaining long-term effects in future trials [5]. Interestingly, besides OC symptoms, several of the above works have documented positive effects on anxiety and depressive symptoms which are frequently encountered in this clinical population [2]. 

In conclusion, most of the available reports suggest beneficial effects of anodal or cathodal tDCS (2 mA) when applied in ten to twenty 20-min sessions over specific frontal lobe areas (OFC, SMA, pre-SMA, DLPFC). However, the current evidence is insufficient and challenged by several limitations mainly related to the paucity of randomized sham-controlled trials, the small sample size of most of them, as well as the heterogeneity in stimulation parameters (different electrodes size and montages), protocol duration, and cohorts’ characteristics. Most of the studies included patients who failed at least one pharmacotherapy (or psychotherapy). Admitting the possible interaction between medications and tDCS, including patients with treatment-naïve OCD would help discerning the potential effects of tDCS independently of other treatments. The available works focused on OCD in adult patients. Therefore, it would be interesting to extend tDCS studies to other age groups (e.g., adolescence, elderly). Moreover, it would be important to control the effects of comorbidities which are frequent in this population in order to judge whether observed effects directly targeted OC symptoms or were mediated by an improvement of other symptoms. Furthermore, some studies have suggested specific neurobiological substrates according to OCD symptoms dimensions [3]. Exploring the latter could increase the current understanding of the matter, and may allow identifying predictors of response to tDCS. There is probably a long way before obtaining sufficient evidence on whether tDCS can join the treatment armamentarium of OCD. Future studies should also focus on selecting the optimal stimulation parameters. Simulating the electric field when designing the studies may allow tailoring the stimulation parameters according to clinical profiles. This might be of relevance since different tDCS montage may be needed according to symptom dimensions. Coupling tDCS with neuroimaging and neurophysiological modalities may help unravel the neural correlates of any observed changes. In addition, admitting the dose-dependent tDCS effects observed in some clinical populations, increasing the number of sessions appears to be pertinent since it may engender long-lasting clinical effects [5]. Besides classical tDCS protocols, research could focus on testing the potential of other types of montages (High definition tDCS) and transcranial electric stimulations (e.g., transcranial alternating current stimulation, transcranial random noise stimulation). Another appropriate issue to consider is when to stimulate. As tDCS effects may depend on the state of the targeted brain circuit [5], it would be logical to ask whether the stimulation should be performed at rest or during task performance (e.g., symptom provocation task). In this perspective, it is worth noting that combining different therapies (medications, psychotherapy, and tDCS) might lead to synergistic effects, which could have its place in more severe OCD cases [11]. Finally, since implementing psychotherapy and tDCS protocol might be difficult to patients and researchers/clinicians, remotely supervised tDCS and online-based CBT interventions have been developed in neuropsychiatry, and upcoming studies would help assess the utility of such a combination in this clinical context [12,13].
